# An ethical analysis of vaccinating children against COVID-19: benefits, risks, and issues of global health equity

**DOI:** 10.12688/wellcomeopenres.17234.1

**Published:** 2021-10-04

**Authors:** Rachel Gur-Arie, Steven R. Kraaijeveld, Euzebiusz Jamrozik

**Affiliations:** 1 Berman Institute of Bioethics, Johns Hopkins University, Deering Hall, 1809 Ashland Avenue, Baltimore, Maryland, 21205, USA; 2 Oxford-Johns Hopkins Global Infectious Disease Ethics (GLIDE) Collaborative, Oxford, UK; 3 Wageningen University & Research, Hollandseweg 1, 6706 KN, Wageningen, The Netherlands; 4 Wellcome Centre for Ethics and Humanities, Big Data Institute, University of Oxford, Li Ka Shing Centre for Health Information and Discovery, Old Road Campus, Oxford, OX3 7LF, UK

**Keywords:** COVID-19 Vaccines, Health Equity, Ethical Analysis, Bioethics, Child Health

## Abstract

COVID-19 vaccination of children over 12 has begun in various high-income countries with regulatory approval and general public support, but largely without careful ethical consideration. This trend is expected to extend to other COVID-19 vaccines and lower ages as clinical trials progress. This paper provides an ethical analysis of COVID-19 vaccination of healthy children. Specifically, we argue that it is currently unclear whether routine COVID-19 vaccination of healthy children is ethically justified in most contexts, given the minimal direct benefit that COVID-19 vaccination provides to children, the potential for rare risks to outweigh these benefits and undermine vaccine confidence, and substantial evidence that COVID-19 vaccination confers adequate protection to risk groups, such as older adults, without the need to vaccinate children. We conclude that COVID-19 vaccination of healthy children in wealthy communities before adults in poor communities worldwide is ethically unacceptable and consider how policy deliberations might evolve in light of future developments.

## Disclaimer

The views expressed in this article are those of the author(s). Publication in Wellcome Open Research does not imply endorsement by Wellcome.

## Introduction

There has been relatively little ethical analysis of vaccinating children against COVID-19. While the approval of a COVID-19 vaccine for children is a testament to rapid scientific progress during the pandemic, applications of scientific achievements are not by default ethically sound ^1^. In this piece, we explore the ethics of vaccinating healthy children against COVID-19, given the minimal direct benefit for children, the potential for rare risks to outweigh such benefits or undermine vaccine confidence, and increasing evidence that COVID-19 vaccination of risk groups such as older adults adequately protects these groups without the need to vaccinate children. We expand our analysis to the global level, highlighting international inequities of COVID-19 vaccine availability and burden of disease. Finally, we discuss potential scenarios in which there could be a stronger ethical case in favor of vaccinating children against COVID-19. We conclude that it is currently difficult to ethically justify the routine vaccination of healthy children, especially given inequitable global vaccine availability.

## Minimal benefit to healthy children

The risks of severe illness in children are much lower than those in older adults, and children are also less susceptible than adults to becoming infected with the virus ^2, 3^. The emergence of the Delta COVID-19 variant has led some scholars to argue that child COVID-19 vaccination is justified, claiming the lower rate of spread and infection among children was not a true representation of infection frequency ^4^. We challenge this claim, for there is currently “no clear evidence that children are more vulnerable to or more affected by Delta compared with earlier variants” ^5^. The burden of COVID-19 in children is similar to or lower than that of typical seasonal influenza during the winter ^6^. Nevertheless, the rare cases of children hospitalized with COVID-19 may be severe, and children with certain severe comorbidities may be at higher risk ^6^. It may well be justifiable to trial and eventually use vaccines routinely in these groups of children, but it is much harder to justify the vaccination of healthy children on the basis of expected benefits to them, given the extremely mild average disease severity. Further, post-infection immunity is at least as effective as vaccination at protecting against (re-)infection in later life, which might otherwise have been more severe ^7, 8^.

It is sometimes claimed that even healthy children frequently experience significant post-acute symptoms (‘long covid’) after mild or asymptomatic infection. However, careful analyses of available data have shown this to be unsupported by current evidence ^9– 11^. Though some COVID illness duration in children is prolonged, research suggests that the burden of symptoms does not increase with time ^12^. Likewise, post-COVID-19 fatigue is strongly correlated with the severity of the acute illness in adults ^13^. Since COVID-19 is generally mild or asymptomatic in healthy children, it is implausible that post-acute symptoms would frequently be severe. Thus, protecting healthy children against long covid does not in itself provide sufficient justification for routine COVID-19 vaccination of healthy children.

Ultimately, unlike many other vaccine-preventable diseases, healthy children are at low risk of COVID-19 morbidity, and mortality ^14^. As long as COVID-19 cases remain on average mild or asymptomatic in children, very large numbers of children would need to be vaccinated to prevent one pediatric COVID-19 hospital admission ^15^. In our view, routine or mandated COVID-19 vaccination of healthy children cannot be justified on the basis of direct individual benefits, and any small, expected benefit may be outweighed by risks and uncertainties.

## Potential risks in children

Policymakers should be especially cautious about potential risks of COVID-19 vaccines in children. First, because avoiding harm to healthy children is arguably key to maintaining vaccine confidence ^16^. Second, because many countries have already recommended against routine use of the AstraZeneca COVID-19 vaccine in children due to concerns that risks might outweigh individual benefits ^17^. Since the expected benefits of COVID-19 vaccination in children are low, the case for routine healthy child vaccination with any COVID-19 vaccine is further weakened by the potential for rare harms. Initial safety data from the clinical trials that motivated the EUA authorization of the Pfizer-BioNTech vaccine for children included 2,260 participants aged 12 to 15, of which 1,131 received the vaccine, and the remainder (1,129) received a placebo ^18^. Recent data suggest that myocarditis is an important rare harm associated with mRNA vaccination, affecting primarily adolescents and young adults, more commonly occurring after the second dose of vaccine, and with a rate in males approximately 10 times that in females ^19^.

Although long-term harms related to vaccines are rare, there should ideally be prolonged follow-up of pediatric trial participants before routine COVID-19 childhood vaccination, especially given the minimal expected direct benefits vaccination would provide for children ^20^. Pfizer released the topline adolescent (12–15 years old) data ^21^, which might at least rule out common adverse events occurring less than eight months post-vaccination. Still, more data are needed. Since relatively few children were involved in the Pfizer-BioNTech clinical trials, and follow-up has not yet been of long duration, regulators are arguably poorly placed to evaluate the risks of rare or delayed adverse outcomes ^22^. Initial data on the long-term outcomes of mRNA-vaccine associated myocarditis, for example, are due to be published in late 2021 ^19^. Moreover, COVID-19 is not a pediatric public health emergency. As a result, we agree with other authors who have suggested that standard criteria for EUA authorization of the Pfizer-BioNTech COVID-19 vaccine for use in children have not been met ^22^.

Historically, vaccine-related harms to children and overly hasty pediatric vaccine rollouts have undermined public confidence in vaccines and cost lives. For example, by the time the risks of a licensed dengue vaccine to a minority of children were confirmed, the Philippines had already vaccinated 830,000 children, many of whom faced increased disease risks if they had never been infected with dengue before ^23^. As a result, at least 130 children died ^24^. Public trust in other vaccines collapsed, leading to local outbreaks of measles and increased risks of other vaccine-preventable diseases ^16, 25^.

Moreover, after the 2009 influenza pandemic, children were identified to be at a greater risk of narcolepsy as a rare vaccine-related harm (five-to-fourteen-fold increase in children as opposed to a two-to-seven-fold increase in adults) ^26^. As a result, some public health authorities suspended the use of this vaccine in children amid public controversy ^27^.

Defects in vaccine production can also pose unintended negative adverse effects in children. Historically, production defects with polio vaccines, for example, were associated with at least one high profile case of harmful outcomes in children, which undermined vaccine confidence at the time ^28^. While the Pfizer-BioNTech vaccine is not a live-attenuated virus, defective production has already occurred for COVID-19 vaccines (e.g., ingredient conflation in 15 million doses of the Johnson & Johnson vaccine) ^29^. Were such defects to go undetected, they might pose harm to vaccine recipients, which, for the reasons outlined above, would be especially concerning in pediatric populations ^30^.

Rare or long-term vaccine-induced risks in children, whether due to defective production or intrinsic to the vaccine but not detected in prior studies, have thus undermined public confidence in the associated vaccines and vaccines in general ^16, 31, 32^. This remained true even when the harms were very rare and if there was an ex-ante net benefit in children. Since it is currently unclear whether mRNA vaccines for COVID-19 are associated with a net benefit in young healthy children, vaccine policy should proceed with caution ^33^. Public trust in vaccines, particularly child vaccines, takes years to build and days to break.

Rather than hastily expanding the use of COVID-19 vaccines in children, a more responsible approach would be to prioritize the systematic collection of pediatric safety and effectiveness data, ideally in placebo-controlled studies. Such studies remain ethical, including from the point of view of equipoise, since it is currently a matter of debate whether the benefits of mRNA vaccines outweigh the risks in healthy children. Such studies could be simultaneous with expansion of access for adolescents with comorbidities in whom the individual benefits more plausibly outweigh risks. If the risks or uncertainties related to mRNA vaccine use in children using standard schedules turn out to be unacceptably high, policymakers could also consider testing single mRNA dose regimens (given that risk of myocarditis appears to increase significantly with the second dose) and/or more widespread use of other types of vaccines (e.g., whole inactivated virus vaccines), which may have a more acceptable safety profile in children ^34^.

## Protecting risk groups does not require vaccinating children

Older adults and other COVID-19 risk groups can be protected without vaccinating children for at least two reasons. First, because children are responsible for a relatively small fraction of transmission in highly vaccinated communities. Second, because COVID-19 vaccines are highly effective against severe disease even where vaccinated individuals are exposed to infection, whether by children or adults.

Data suggest that children are less susceptible to developing SARS-CoV-2 infection and, even if infected, are less likely to infect others as compared to adults ^35^. The majority of secondary infections directly attributable to children occur within households. If adult household members are vaccinated, the risks to the wider community may be reduced still further ^36, 37^. Though controversy persists regarding the effect of school closures on COVID-19 community transmission, recent data suggest that reopening schools does not result in large resurgent epidemics of severe disease in populations where a large proportion of adults are vaccinated ^37, 38^. In sum, children are not major vectors of COVID-19 transmission, insofar as their risks of becoming infected are lowered further by high rates of adult vaccination.

Perhaps the strongest case against child COVID-19 vaccination is the fact that COVID-19 vaccines are safe and effective in higher-risk groups, including older adults and the immunocompromised ^20, 39^. Ethical arguments in favor of vaccinating healthy, young people for the benefit of others in the context of other diseases generally emphasize that the generation of population (“herd”) immunity is one of the only ways to protect vulnerable risk groups ^40^. Such risk groups may be unable to generate a sufficient immune response to other vaccines, such as those for influenza and pneumococcus ^41– 43^. In these cases, risk groups might depend on healthy children to get vaccinated in order to protect others. This is not the case for COVID-19. Risk groups experience highly effective protection against severe COVID-19 from currently available vaccines, even if they are exposed to significant community transmission ^44^. As a result, the ethical claim that young, healthy children should get vaccinated especially for the benefit of risk groups – which might constitute a strong argument in favor of the vaccination of healthy children against other diseases – does not hold in the case of COVID-19 vaccines ^1, 45^.

Overall, the contribution to population-level immunity per child vaccinated is significantly lower than the contribution per adult vaccinated. Inadequate vaccination of adult populations should not be an excuse for the vaccination of children. Furthermore, it would be poorly cost-effective and ethically unjustified to use children as a means to protect vaccine-hesitant adults - especially if vaccinating children involves non-trivial risks.

Once a high proportion of adults are vaccinated, if residual transmission levels and disease burden are deemed unacceptable, COVID-19 vaccination of children might, under certain conditions, produce additional reduction in these outcomes. Such conditions may include higher risk individuals who happen to be poorly protected by vaccines remaining at risk by exposure to otherwise-healthy children infected with COVID-19. Until adult vaccination uptake in communities is maximized, it is not clear that it would be ethically justifiable to promote COVID-19 vaccination of children to boost population immunity. Moreover, since current COVID-19 vaccines do not provide sterilizing immunity, and since post-vaccination infections are relatively common, no amount of community vaccination will produce elimination of transmission. Vaccinated adults will be infected sooner or later. While higher rates of child vaccination might delay infections in some adults to some degree, these infections cannot be prevented. The ubiquity of post-vaccination infections and re-infections therefore undermines the weight of any ethical argument to the effect that vaccinating children is required to prevent harm to others.

## Perpetuation of global vaccine inequities

The COVID-19 pandemic has been characterized by domestic and international inequities ^46^, including but not limited to inequities in the distribution of the burdens of disease, scarce healthcare resources, social and economic opportunities, and, most recently, vaccines ^47^. As of April 2021, 700 million COVID-19 vaccine doses had been globally administered, with high income countries (HICs) securing 87% of doses, and low-income countries (LICs) securing only 0.2% of doses ^48^. These inequities are further exacerbated by the administration of ‘booster’ vaccines in HICs, while LMICs remain without first and second doses. Israel now administers ‘booster’ doses to all eligible individuals five months after their second dose ^49^, while Germany, France, and the United States anticipate boosting all eligible individuals from September 2021 ^50, 51^. Though many HICs have committed to donate COVID-19 vaccine doses to low- and middle-income countries (LMICs), these donations are only a small fraction of the 1.8 billion dose goal of COVAX and are not a sustainable solution to equitably vaccinating higher risk individuals worldwide ^52^.

Vaccinating adults in LMICs alongside those in HICs is arguably the best way of ensuring global equity and ensuring positive child health and wellbeing outcomes ^53^. Adults are often the key support for one or more children and other vulnerable populations, such as older adults ^54^. When that support system is threatened, either through illness or death, children suffer socially and economically. As a result, children may have to stop attending school, or other social services, in order to work -- or may be forced into child marriages, which have increased in frequency during the pandemic ^55^. Accordingly, the social benefit of vaccinating adults, particularly in LMICs, far outweighs the benefit of vaccinating healthy children in HICs ^45^. As a global community, we should focus on vaccinating adults in LMICs – beginning with healthcare workers and older adults and lowering the age as supply increases – rather than healthy children in HICs.

## COVID-19 vaccine policy and potential future developments

Ethical evaluations of routine childhood vaccination against COVID-19 are, of course, subject to future developments. First, international travel has not returned to pre-pandemic levels (and many countries’ borders have been closed or entry restricted) ^56^. An increase in travel might increase transmission - though not necessarily affecting disease burden to the same degree, provided that vaccines and post-infection immunity remain effective in the prevention of severe disease. Second, the UK and Israel have a high level of population immunity due to past infection, whereas countries that largely eliminated COVID-19 (for example Australia, New Zealand, or Taiwan ^57^) might require even higher rates of adult vaccination to achieve similar levels of disease control. Third, vaccine-derived immunity may wane over time, especially where individuals are not re-exposed to infection soon after vaccination.

As a result, one might think that there would be contexts in which child COVID-19 vaccination would be more ethically acceptable on a routine basis than is currently the case. However, some factors leading to unacceptable disease burdens could be mitigated with other measures. For example, waning immunity could be controlled by repeat ‘booster’ COVID-19 vaccination of vulnerable adults ^58^. Moreover, since SARS-CoV-2 will likely become globally endemic ^59^, causing mild childhood illness akin to other seasonal human coronaviruses, post-infection immunity in children will also make a positive contribution to disease control. The case for vaccinating children might therefore become even weaker in the long term since post-infection immunity will be well-matched to circulating variants and robust across closely related variants as is known to be the case for other coronaviruses ^8, 60^. Post-infection immunity in children might thus be expected to provide more sustainable public health benefits than a reliance on regular updates of vaccines.

Furthermore, there is increasing evidence that COVID-19 vaccines do not altogether prevent infection ^61^. This creates a concern that by vaccinating children, infection may only be delayed (through waning vaccine efficacy), thus instigating the potential need for ‘booster’ shots. While the risk-benefit profile of offering ‘boosters’ to groups at high risk may turn out to be favorable, this is unlikely to be the case for children, given the previously discussed low net benefits of COVID-19 vaccination and the potential for any harms to outweigh those benefits.

Nevertheless, should high adult vaccination uptake be insufficient to control disease burden – as opposed to mere transmission of the virus – a stronger case for routine child COVID-19 vaccination could emerge. Despite mainstream public health officials suggesting otherwise, and even going so far as to advocate for mandatory child COVID-19 vaccination ^62^, the Delta variant currently does not threaten the feasibility of high adult COVID-19 vaccination uptake to control outbreaks. As a result, the ethicality of routine COVID-19 vaccination of healthy children is currently weak – and the case for mandating even weaker. Additionally, the ethical acceptability of routine child COVID-19 vaccination under any circumstances is contingent on reliable long-term safety data from clinical trials involving children. Should severe side effects arise in healthy children, even if they are rare, the overall public health harms of vaccinating healthy children, including reductions in vaccine confidence, may outweigh any benefits.

Ultimately, parents and guardians of children should weigh the potential risks and benefits of vaccination in their own context. Currently, every vaccine used for a healthy child in a wealthy community would most likely be better used for an adult at high risk of severe illness from COVID-19 in a poor community. Perhaps in the future, should global vaccine supply be sufficient to meet demand, and should more data become available regarding the long-term safety of COVID-19 vaccines in children, ethical considerations might weigh in favor of COVID-19 vaccination of children, without making COVID-19 vaccines routine or mandatory for all children. At present, routine vaccination of healthy children against COVID-19 on balance, to be unjustified.

## Data availability

No data are associated with this article.
