# Supplementary material for: An ethical analysis of vaccinating children against COVID-19: benefits, risks, and issues of global health equity
Source: Wellcome Open Res. 2021 Nov 29;6:252. Originally published 2021 Oct 4. [Version 2] doi: 10.12688/wellcomeopenres.17234.2 (PMC11496933; doi:10.12688/wellcomeopenres.17234.2)
Supplement: Peer review history

# Reviewer report: Reviewer response for version 2

**DOI:** 10.21956/wellcomeopenres.19290.r106527

**Published:** 2024-12-06

**Authors:** Ming-Jui Yeh

**Affiliations:** 1 Institute of Health Policy and Management, National Taiwan University, Taipei City, Taipei City, Taiwan

December 5, 2024

 This Open Letter challenges the ethical acceptability of routine and/or mandatory COVID-19 vaccination for children at the global level. In general, I found the arguments and the corresponding policy suggestions reasonable. Below, I offer some further thoughts for the authors and readers to consider. I noticed that this article was written in the context of the early stage of the COVID-19 pandemic in around 2021; the evidence and knowledge we have now are quite different from then. Therefore, I do not comment on the empirical part, only on the ethical arguments.

 First, the authors' ethical arguments largely (if not entirely) rely on the utilitarian account, i.e., the effectiveness of doing or not doing any interventions/policies, which further depends on the latest evidence regarding the pandemic itself and the development and trial outcomes of vaccine products. However, the authors do not make this main ethical ground clear in any place in the article. I would suggest that the authors make their ethical grounds clear up front in the analysis.

 Second, the authors’ responses to other reasonable objections or concerns are also largely utilitarian. There are likely reasonable challenges to their arguments from other ethical perspectives/approaches, for instance, from the perspectives of protecting human rights to health, care ethics, and relational autonomy, among others.

 Third, the authors could consider providing a list of the empirical conditions for their arguments to be valid, for instance, that there is a prevalent vaccine shortage (as the authors have mentioned in the last paragraph), that the vaccines are less effective for children than adults, and that the adverse events on children would cause the public more vaccine hesitated sentiments than adults. If any of the conditions have changed now or would change in the future, the readers could get a quick sense of the limitations of the arguments.

 Fourth, even if the readers would agree with the empirical conditions and agree with the author's utilitarian arguments, one could still challenge why children matter. Consider the authors' definition; they focus on "young, healthy children (e.g., those aged under 15 with no comorbidities)," but why 15, not 18? According to international human rights covenants, the general cut-off age for children is 18. Even if the authors would revise their definition to 18, we could further challenge, why not 20? As most of the authors’ arguments are based on empirical populational level evidence, there would be a problem with the arbitrary choice of the cut-off age. Those populations under 15, 18, or 20 (young adults) are likely to show similar patterns as compared to older adults, making most of the authors’ arguments valid.

 Fifth, the authors do slightly mention equity, which is different from utilitarianism in the allocation of vaccines at the global level. However, the transitions of the argument in that section are embedded with logical leaps. The authors appeal to "[a]s a global community" (p. 5) as the reason for prioritizing vaccines for adults (in LIMCs) rather than children (in HICs). This is not sufficient to argue for global allocation in general, not to mention global allocation between specific subpopulations (e.g., adults in LIMCs and children in HICs).

Does the article adequately reference differing views and opinions?

No

Are all factual statements correct, and are statements and arguments made adequately supported by citations?

Partly

Is the Open Letter written in accessible language?

Yes

Where applicable, are recommendations and next steps explained clearly for others to follow?

Yes

Is the rationale for the Open Letter provided in sufficient detail?

Yes

Reviewer Expertise:

public health ethics

I confirm that I have read this submission and believe that I have an appropriate level of expertise to confirm that it is of an acceptable scientific standard, however I have significant reservations, as outlined above.

# Reviewer report: Reviewer response for version 2

**DOI:** 10.21956/wellcomeopenres.19290.r110505

**Published:** 2024-12-04

**Authors:** Seema Shah, Jeffrey Poomkudy

**Affiliations:** 1 Feinberg School of Medicine, Northwestern University - Chicago (Ringgold ID: 205058), Chicago, Illinois, USA; 2 Northwestern University Feinberg School of Medicine, Chicago, Illinois, USA

We appreciate the opportunity to review this well-written and thought-provoking paper. The article raises several issues that are worth considering, and the article lays out an interesting argument prioritizing the vaccination of adults globally over routine COVID-19 vaccination in children in higher income countries. However, we have a number of suggestions to improve the article that are outlined below:

 1. Some parts of the paper are a bit outdated. While this may be a result of when the paper was written, submitted, and published, the authors should consider updating the claims made in the paper for posterity, or by noting the time at which these claims held true. For example:

- The authors suggest that there is not enough data to prove the safety of COVID-19 vaccination in children. With the accumulated evidence from national surveillance (see, for instance: https://www.nature.com/articles/s41467-024-47745-z and https://pmc.ncbi.nlm.nih.gov/articles/PMC9706403/), it should now be clear that there is substantial evidence demonstrating the safety of COVID-19 vaccines in children. The incidence of adverse effects, including pericarditis and myocarditis, are very rare and concentrated in male adolescents after their second dose.
- The article also notes that “international travel has not returned to pre-pandemic levels (and many countries’ borders have been closed or entry restricted).” Although international travel has not quite returned to pre-pandemic levels as of current writing (though very close), countries have re-opened their borders.

2. Second, and relatedly, we believe that the risk-benefit analysis of COVID-19 vaccination is flawed and may benefit from some refinement based on the most recent evidence, as noted above. 80% of MIS-C cases in 2023 were in unvaccinated children. Absolute numbers of MIS-C are considerably lower than before, which the CDC speculates is due to immunity from vaccination and prior infection, as well as a change in the case definition for MIS-C in 2023 from 2020 (see: https://www.cdc.gov/mmwr/volumes/73/wr/mm7310a2.htm). It is hypothesized that vaccination is critical to keep the number of cases of MIS-C low. Given the rare adverse events associated with COVID-19 vaccination, the health benefit of avoiding severe COVID-19 and MIS-C would seem to outweigh these risks in most children.

 Additionally, indirect benefits also ought to be considered in the cost-benefit analysis. Unvaccinated children may put immunocompromised parties at risk, especially individuals who may not be able to mount an adequate immune response, even after receiving booster doses (see: Parker, E. P. K., Desai, S., Marti, M., Nohynek, H., Kaslow, D. C., & Kochhar, S. Response to additional COVID-19 vaccine doses in people who are immunocompromised: a rapid review. *Lancet Glob Health*. 2022; 10 (3): e326–8). Though COVID-19 vaccination does not provide sterilizing immunity, it reduces the likelihood of developing more severe disease, which in turn may decrease overall transmissibility. There is evidence that infections after vaccination may reduce transmissibility in households. See: Harris, R. J., Hall, J. A., Zaidi, A., Andrews, N. J., Dunbar, J. K., & Dabrera, G. (2021). Effect of vaccination on household transmission of SARS-CoV-2 in England. *New England Journal of Medicine*, 385(8), 759-760. Earlier in the pandemic, the delay in vaccinating children contributed to prolonged school closures in the U.S, as teachers in some parts of the country were concerned about the safety of reopening when children were unvaccinated. Prolonged school closures caused substantial learning losses and exacerbated a mental health crisis. (See, e.g., https://www.chalkbeat.org/chicago/2021/8/12/22622602/covid-19-school-quarantine-debate-unfolds-chicago-teachers-union-ctu-cps-classroom-closures/; https://jamanetwork.com/journals/jamanetworkopen/fullarticle/2821950#:~:text=Transitioning%20from%20online%20to%20in,18%25%20increase%20in%20adolescent%20suicide.&text=Conversely%2C%20there%20are%20also%20suggestions,with%20worse%20adolescent%20mental%20health.) Unvaccinated children also faced longer periods of isolation. It is also important to note the public health costs associated with further mutation from uninhibited spread in a susceptible population.

 3. We agree with the authors that global health equity is critically important. But we wonder how relevant this issue is now, as global vaccine inequities are now ameliorated ( https://www.nytimes.com/interactive/2021/world/covid-vaccinations-tracker.html and Nohynek, H., & Wilder-Smith, A. (2022). Does the world still need new Covid-19 vaccines? *New England Journal of Medicine*, 386(22), 2140-2142.) The argument had much more force at a certain point in the pandemic, and it may be relevant for future pandemic preparedness for the authors to be more precise about when the needs of adults globally might outweigh the interests of children in higher income countries.

 Also, the authors presume an ethical framework for global justice without making it explicit. We assumed they are situating their argument within a cosmopolitan framework, in which countries have similar ethical obligations to individuals living outside their borders as they do to their own citizens. Yet there was a robust debate about global justice frameworks, with some strong arguments for moderate nationalism (see: Lie, R. K., & Miller, F. G. (2020) Allocating a COVID‐19 vaccine: balancing national and international responsibilities. *The Milbank Quarterly*, 99(2), 450)). Moreover, the global trend politically appears to be moving towards more nationalistic and isolationist tendencies, which seems important for the authors to acknowledge. It is critical that the authors clarify their stance. It would also be helpful if they argued for their position, or at least cited some literature in support of their view if substantiation of that stance is beyond the scope of their paper. (See, e.g., https://jamanetwork.com/journals/jama/fullarticle/1905876)

 It also may be worth noting the complexity of transferring vaccines from HIC to LMIC. As mentioned above, there may now be a more limited need for vaccines in LMICs. There is also a practical dimension to consider. When vaccines were donated, they were often donated when they were about to expire, leading to waste (see: Lazarus, J. V., Karim, S. S. A., Van Selm, L., Doran, J., Batista, C., Amor, Y. B., ... & Yadav, P. (2022). COVID-19 vaccine wastage in the midst of vaccine inequity: causes, types and practical steps. *BMJ Global Health*, 7(4), e009010). Some of logistical considerations include the dosing differences between pediatric and adult populations, as some pediatric doses come in pre-filled syringes, and some manufacturers have different concentrations in different vials ( https://www.cdc.gov/vaccines/covid-19/downloads/covid-19-immunization-schedule-ages-6months-older.pdf). For some of the politics that complicate vaccine donation, see: de Bengy Puyvallée, A., & Storeng, K. T. (2022). COVAX, vaccine donations and the politics of global vaccine inequity. *Globalization and Health*, *18*(1), 26. This is empirical data that may be worth noting. Perhaps this is not something the authors want to contend with, in which case they should note that they are taking an ideal theory perspective.

 4. The authors note that errors in vaccine production would disproportionately undermine trust in vaccination and affect the uptake of childhood vaccination. This is undoubtedly true, but it holds for all other vaccines. Therefore, its relevance to the argument presented in the paper is low and they should consider deleting this statement.

 5. The authors state that “the burden of COVID-19 in children is similar to or lower than that of typical seasonal influenza during the winter.” This seems to cast at least some doubt on the need for routine vaccination of children for influenza. If the authors are arguing against routine COVID-19 vaccination in children, then they should contend with what that means for childhood vaccination against influenza or explain how to distinguish the two. Their argument has the most force regarding the relative benefit of vaccination for adults versus children, and the article would be stronger if the authors maintained focus on this claim.  

 6. The authors state that “Recent data suggest that myocarditis is an important rare harm associated with mRNA vaccination, affecting primarily adolescents and young adults, more commonly occurring after the second dose of vaccine, and with a rate in males approximately 10 times that in females.” If the primary adverse events occur almost exclusively in males between 12-30, within 1-14 days post vaccination after the second pose of the primary series of mRNA vaccines, then the argument to limit vaccines ought not to extend beyond this group. The authors should consider restricting their argument to the relevant group.

 7. The authors argue that “As long as COVID-19 cases remain on average mild or asymptomatic in children, very large numbers of children would need to be vaccinated to prevent one pediatric COVID-19 hospital admission. ^17^” However, the article they cite does not support this claim, as the article does not suggest that a certain number of children would need to be vaccinated in order to prevent one pediatric COVID-19 hospitalization. The article *does *support the substantive part of the first clause, that COVID-19 is mostly mild in children.

 8. The authors state at the end of the article, “Perhaps in the future, should global vaccine supply be sufficient to meet demand, and should more data become available regarding the long-term safety of COVID-19 vaccines in children, ethical considerations might weigh in favor of COVID-19 vaccination of children.” As has been stated, safety has been established after years of surveillance, and global vaccine supply has met demand. It is worth reframing this statement in light of these new developments, especially if the authors want to maintain opposition to the routine vaccination of children against COVID-19.

 9. Finally, one minor point is that the authors argue that: “we agree with other authors who have suggested that standard criteria for EUA authorization of the Pfizer-BioNTech COVID-19 vaccine for use in children have not been met.” EUA criteria require the declaration of a public health emergency ( https://www.fda.gov/emergency-preparedness-and-response/mcm-legal-regulatory-and-policy-framework/emergency-use-authorization). This article has a nice explanation of how the standard for EUA is relatively low, and the question was whether the benefits of vaccination outweighed the risks: https://pmc.ncbi.nlm.nih.gov/articles/PMC11034974/. Unless the authors would like to provide a more solid defense of this point, it may be better to delete the sentence.

Does the article adequately reference differing views and opinions?

Partly

Are all factual statements correct, and are statements and arguments made adequately supported by citations?

Partly

Is the Open Letter written in accessible language?

Yes

Where applicable, are recommendations and next steps explained clearly for others to follow?

Yes

Is the rationale for the Open Letter provided in sufficient detail?

Partly

Reviewer Expertise:

Global health research ethics, pediatric research ethics

We confirm that we have read this submission and believe that we have an appropriate level of expertise to confirm that it is of an acceptable scientific standard, however we have significant reservations, as outlined above.

# Reviewer report: Reviewer response for version 2

**DOI:** 10.21956/wellcomeopenres.19290.r110506

**Published:** 2024-12-03

**Authors:** Malik Sallam

**Affiliations:** 1 The University of Jordan, Amman, Jordan

Thanks for the invitation to review this timely, excellent, and relevant manuscript.

 In this letter, Rachel Gur-Arie *et al*. presented a comprehensive evaluation of the ethical aspects associated with vaccinating children against COVID-19. The authors provided a balanced argument on the necessity of a thorough re-evaluation of these ethical aspects, emphasizing both the collective benefits and the potential risks to children which is a concern justified by the lack of current strong evidence to fully support this approach as an established practice.

 The discussion presented in this manuscript carefully addressed nearly every critical angle of the ongoing debate on the ethical aspects regarding vaccinating healthy children against COVID-19. The manuscript examined the benefits of vaccinating healthy children, the associated risks, global vaccine inequities, and the implications for future COVID-19 vaccine policies. The authors’ approach was thorough and helps to understand the complexities involved in this controversial topic.

 The letter is well-written and comprehensive. Therefore, I fully endorse it for publication given the soundness of the content. Additionally, I feel sorry for the extended duration it took for the peer review and approval process since this work deserved swifter attention!

**Minor suggestions:**

- In the Introduction: The sentence “We conclude that it is currently difficult to ethically justify the routine vaccination of healthy children, especially given inequitable global vaccine availability” can benefit from explicitly mentioning that it refers to COVID-19 vaccinations to avoid any potential misunderstanding regarding generalization to the established childhood vaccines.
- Under the section “Protecting risk groups does not require vaccinating children”: The statement “We reject this claim. Older adults and other COVID-19 risk groups can be protected without vaccinating children for at least two reasons” could benefit from revising the phrasing. The assertive language in the statement might be perceived as dismissive rather than analytical. However, this is only a suggestion for improvement and the authors should not feel obliged to revise it based on my comment!

Best wishes

Does the article adequately reference differing views and opinions?

Yes

Are all factual statements correct, and are statements and arguments made adequately supported by citations?

Yes

Is the Open Letter written in accessible language?

Yes

Where applicable, are recommendations and next steps explained clearly for others to follow?

Yes

Is the rationale for the Open Letter provided in sufficient detail?

Yes

Reviewer Expertise:

Vaccination attitude; vaccine hesitancy

I confirm that I have read this submission and believe that I have an appropriate level of expertise to confirm that it is of an acceptable scientific standard.

# Reviewer report: Reviewer response for version 2

**DOI:** 10.21956/wellcomeopenres.19290.r111947

**Published:** 2024-11-30

**Authors:** Saurav Basu

**Affiliations:** 1 Community Medicine, ESIC Medical College & Hospital, Kolkata, West Bengal, India

The authors argue against mass vaccination of children against Covid-19 based on certain importaant public health considerations - primarily the lack of pronounced benefit for both individual children and the community.

 Arguments of potential side effects of Covid-19 vaccines in children potentially undermining public trust of vaccines used even for routine immunization are true but can be overcome by generation of evidence indicating vaccine safety by the manufacturers. Hence, the authors could perhaps focus on whether there is any necessity of vaccination even if these Covid-19 vaccines are found to be absolutely safe for use in children. The cost-effectiveness of vaccinating children against Covid-19 is minimal and this should be emphasized.

 If herd immunity can be achieved in children even in absence of vaccination, then the argument for mass and mandatory vaccination is sufficiently attenuated. 

 Evidence from LMICs especially India can be considered in this regard where there is ample evidence that most children (81.8%) with progression of the Covid-19 pandemic had natural infection induced immunity in absence of government mandate for pediatric Covid-19 vaccination.

 https://pubmed.ncbi.nlm.nih.gov/35820667/

Does the article adequately reference differing views and opinions?

Partly

Are all factual statements correct, and are statements and arguments made adequately supported by citations?

Yes

Is the Open Letter written in accessible language?

Yes

Where applicable, are recommendations and next steps explained clearly for others to follow?

Partly

Is the rationale for the Open Letter provided in sufficient detail?

Yes

Reviewer Expertise:

Public health research, public health ethics,  infectious disease and NCD epidemiology

I confirm that I have read this submission and believe that I have an appropriate level of expertise to confirm that it is of an acceptable scientific standard, however I have significant reservations, as outlined above.

## References

[1] : Seroprevalence of immunoglobulin G antibodies against SARS-CoV-2 in children and adolescents in Delhi, India, from January to October 2021: a repeated cross-sectional analysis. *Osong Public Health Res Perspect* .2022;13(3) : 10.24171/j.phrp.2022.0014 184-190 10.24171/j.phrp.2022.0014 35820667 PMC9263332

# Reviewer report: Reviewer response for version 2

**DOI:** 10.21956/wellcomeopenres.19290.r104158

**Published:** 2024-10-22

**Authors:** Y. M. Barilan

**Affiliations:** 1 Tel Aviv University, Tel Aviv-Yafo, Israel

This is a well written paper. However, there need be stronger distinction between ethical and prudential considerations. Weather there is benefit to the public is a question for public health experts. Weather beneficial vaccinations (whoever may be benefited) are justified despite considerations of justice, respect for persons etc., is an ethical question. If vaccinating children is useless, there is no ethical question here.

 This must be acknowledged throughout the discussion. For example, perhaps herd immunity by children vaccination is key to successful vaccination of adults. Hence the argument from justice may not work. Isn't there a risk that vaccination of adults in the rich countries plus vaccination of some in LIMC countries my result in dual failure? I dont know. I am an ethicist. But the authors should consider such questions. They also should show awareness of the incompatibility of their arguments. If children vaccination is more harmful overall, we need not the justice consideration., Only if vaccination of children is good, but doling the vaccines out to the poor does better, the ethical question becomes really intriguing. 

 (If laws of intellectual property change, we may be able to do both. In that case, the tension between the good (even if very minor) of vaccinating children and the greater good of the poor is illusory.

 The last two sentences of the article embody this self-contradictory way of argumentation. It is always true that people should consider their own risk-benefit balance in healthcare decisions. This consideration is independent of the justice question. One may wonder what should a conscientious person do if the risk to self or child is significant but very tiny in absolute terms, but the benefit to others is no less significant but of much more benefit to the vulnerable. 

 This may lead to an apparent absurd: Perhaps we should vaccinate (i.e. recommend and support) only children in LMIC, because in many such countries children comprise of huge proportion of the population. Almost half! of Gaza's population is children... Might it be possible that the only way to confer herd immunity on such societies is to include children in the vaccination programs?

 The authors should also differentiate generic from specific issues. Worries about faulty production is relevant to any medicine and product. Unless they show us that this worry is different in the context of covid vaccination, the point seem superfluous. 

 Some language needs moderation. "rather than hastily expanding...". Well this is the conclusion, not part of the argument. Moreover, so far the discussion touched principled issues, not the speed of vaccine introduction. The authors confuse the reader: are they leading in the direction of mitigation (more gradual introduction, better selection of recipients, or a principled one - to to vaccinate at all, at least before the rich are vaccinated.

 I also wish the authors acknowledge the absolute low risk of complications. We expose children to much higher risks all the time - road accidents on the way to school (if you chose to live near busy roads), unhealthy eating habits, sports injuries in tooball etc. etc. etc. If vaccination of children is of some social value, what is the ethical argument against exposure to the same level of risk and less, in the benefit of society, and possibly some vulnerable relatives?

 Perhaps the question of vaccination may be considered from the perspective of over-prescription of antibiotics and xrays and many other medical procedures, "Just to be on the safe side"'  (See Makary's 2024 book "blind spots" ch. 3. All such antibiotics will serve LIMC better as well. Perhaps the issue at stake is not limited to vaccination, but a different one?

 Personally, I am quite troubled by the risk that my child transfer the virus to a kidney transplanted (for example). Isn't it in the great benefit of children that they do not become carriers of risks to others?

 Isn't it my moral responsibility?

 I wonder. Benefit is not only a material benefit, perhaps,. 

 Some important references are missing:

 Offit's editorial in JAMA Pediatrics, January 2023. 

 Brusa and Barilan, Journal of Bioethical Inquiry, 2022.

 and

 Bardosh....Makary et al. in Journal of Medical ethics, 2024. 

 The latter is not exactly the same kind of population but worth reading. 

Does the article adequately reference differing views and opinions?

Partly

Are all factual statements correct, and are statements and arguments made adequately supported by citations?

Yes

Is the Open Letter written in accessible language?

Yes

Where applicable, are recommendations and next steps explained clearly for others to follow?

Yes

Is the rationale for the Open Letter provided in sufficient detail?

Yes

Reviewer Expertise:

medical ethics and history, internal medicine

I confirm that I have read this submission and believe that I have an appropriate level of expertise to confirm that it is of an acceptable scientific standard, however I have significant reservations, as outlined above.

# Reviewer report: Reviewer response for version 2

**DOI:** 10.21956/wellcomeopenres.19290.r92210

**Published:** 2024-08-26

**Authors:** Kumanan Wilson

**Affiliations:** 1 University of Ottawa, Ottawa, ON, Canada

This is an important topic to discuss and a challenging one.  

 This paper can be valuable however it needs more clarity.

**My concerns are as follows:**

 1.  I still remain unclear as to what ethical framework was used for this analysis.  The paper simply states global public health ethics framework but doesn't describe this.

 There are clear principles of medical ethics and of public health ethics.  Which ever is used these should be described.

 2.  The paper seems to lump together routine and mandatory vaccination of children.  These are considerably different actions with substantially different ethical analyses.  If the paper is primarily referring to routine voluntary vaccination, it appears that the authors are arguing individuals shouldn't be able to vaccinate their children if they want to given transparent communication, although the conclusion seems to walk this back a bit.  Really the paper isn't make an ethical argument but a scientific one that the benefits of the vaccine don't outweigh the risks.  The argument against mandatory vaccination is clearer.

 3.  When discussing vaccination requirements for children to benefit others it would be important to discuss the difference between sterilizing and protective immunity.  The latter would argue in favor of individuals making the choice for the protection of their own children.  Arguably this may be necessary to also protect the health system if it is being overwhelmed by cases.  In the case of sterilizing immunity the argument could be made that vaccinating a child could potentially protect others.  My understanding is that the evidence for robust, long lasting sterilizing immunity is weak which is at the heart of why this argument is problematic.  This is alluded to towards the end of the article and would be better discussed earlier.

 4.  In cases were vaccines are required or strongly recommended it would be important to mention reciprocal responsibilities of the state to the individual - informed consent, AEFI surveillance systems, vaccine injury compensation for example.

 5.  Ultimately, I think this paper is really about global distributive justice and I think it would be best if its framed from this perspective.  This argument holds if their continues to be a scarcity of the vaccine which I am not sure there still is.

 6.  This paper seems to be written with Delta in mind.  A lot has changed since then - severity of COVID, availability of vaccines, science on the effectiveness of vaccines.  Maybe using COVID-19 as an example for future ethical challenges would be better.  The current m-pox public health emergency points to this as some higher income countries may be unwilling to share their vaccines with lower income countries who could derive more benefit from the vaccine.

Does the article adequately reference differing views and opinions?

Yes

Are all factual statements correct, and are statements and arguments made adequately supported by citations?

Partly

Is the Open Letter written in accessible language?

Yes

Where applicable, are recommendations and next steps explained clearly for others to follow?

Partly

Is the rationale for the Open Letter provided in sufficient detail?

Partly

Reviewer Expertise:

Immunization and health policy

I confirm that I have read this submission and believe that I have an appropriate level of expertise to state that I do not consider it to be of an acceptable scientific standard, for reasons outlined above.

# Reviewer report: Reviewer response for version 1

**DOI:** 10.21956/wellcomeopenres.19048.r46337

**Published:** 2021-11-01

**Authors:** Tracy Beth Høeg

**Affiliations:** 1 Department of Physical Medicine & Rehabilitation, University of California-Davis, Sacramento, CA, USA

This is an excellent letter. It is very important and timely. The only things I recommend the authors consider would be:

1. Making clearer what the current argument for vaccinating all children is - such as acknowledging there have indeed been deaths among healthy children and that MIS-C also poses a threat to healthy children. I think addressing these specific issues will make the letter stronger.
2. It would be good to put the COVID-19 vaccine in the context of other routine childhood vaccinations - why they are recommended for children and how the amount of safety data we have on those differs from what we have the COVID-19 mRNA vaccines. 
3. Consider mentioning the suspension of Moderna in younger age groups in Nordic countries. 
4. Please also consider mentioning the much smaller potential benefit to children who are already immune and how the risk of the vaccination would most certainly outweigh the benefit in most of these children.

Thank you. I commend all of the authors on the excellent work.

Does the article adequately reference differing views and opinions?

Partly

Are all factual statements correct, and are statements and arguments made adequately supported by citations?

Yes

Is the Open Letter written in accessible language?

Yes

Where applicable, are recommendations and next steps explained clearly for others to follow?

Yes

Is the rationale for the Open Letter provided in sufficient detail?

Yes

Reviewer Expertise:

Epidemiology and Public Health

I confirm that I have read this submission and believe that I have an appropriate level of expertise to confirm that it is of an acceptable scientific standard.

# Response

**Published:** 2021-11-22

**Authors:** Rachel Gur-Arie

**Affiliations:** Johns Hopkins University, USA

This is an excellent letter. It is very important and timely. The only things I recommend the authors consider would be:

 Making clearer what the current argument for vaccinating all children is - such as acknowledging there have indeed been deaths among healthy children and that MIS-C also poses a threat to healthy children. I think addressing these specific issues will make the letter stronger.

- Thank you for this suggestion. We have incorporated this point into the ‘minimal benefit to healthy children’ section: “With this in mind, healthy children can suffer significant adverse effects from COVID-19. A multisystem inflammatory syndrome (MIS-C) has been associated with COVID-19 among healthy children, and healthy children have died from COVID-19. Every child death is a tragedy. Still, current data suggests these cases to be a small minority of COVID-19 cases among healthy children.” We have also added two references.

It would be good to put the COVID-19 vaccine in the context of other routine childhood vaccinations - why they are recommended for children and how the amount of safety data we have on those differs from what we have the COVID-19 mRNA vaccines. 

- Excellent point, thank you. We have added in an example of the chickenpox vaccine in the ‘“potential risks to children” section.

Consider mentioning the suspension of Moderna in younger age groups in Nordic countries. 

- Thank you for this suggestion! We have added this in to the “potential risks in children section and added the reference: Paterlini, M. (2021). Covid-19: Sweden, Norway, and Finland suspend use of Moderna vaccine in young people “as a precaution”. BMJ.

Please also consider mentioning the much smaller potential benefit to children who are already immune and how the risk of the vaccination would most certainly outweigh the benefit in most of these children.

- Thank you for suggesting incorporating this important nuance. We have included it in the “potential risks to children” section: “The small potential benefit to healthy children who have post-infection immunity to COVID-19 would most likely be outweighed by the potential risks of COVID-19 vaccination.”

Thank you. I commend all of the authors on the excellent work.

- Thank you for reviewing our manuscript!

# Reviewer report: Reviewer response for version 1

**DOI:** 10.21956/wellcomeopenres.19048.r46336

**Published:** 2021-10-18

**Authors:** Stefan David Baral

**Affiliations:** 1 Department of Epidemiology, Johns Hopkins Bloomberg School of Public Health, Baltimore, MD, USA

This article is focused on providing a synthesis of the ethics of COVID-19 vaccination for children. Overall, I think an evidence-based synthesis that it remains unclear even within communities to vaccinate all young children with the currently available vaccines and also that it is particularly challenging in the context of global vaccine inequities.

 The strengths are clear in that it takes a neutral tone and is data-driven. However, I believe it could be strengthened in a few ways.

 Since it is not a systematic review, I believe the way to maximize the potential impact of the work is to present it in the context of a broadly accepted ethical framework. It may be a single framework that evaluates the individual risk:benefit ratio of vaccination and then another one that evaluates it globally--or maybe the same framework if feasible. Either way, I think presenting it this way will go a long way in terms of the transition from this being a commentary to more of an ethical analysis. At the individual level, I also think including the argument that is often used about even if there is some risk to children, the benefit of decreased onward transmission is worth it--i.e., that children potentially assume risk for population-level benefits. It would be helpful that the ethical analysis includes an assessment of this within broadly accepted ethical frameworks.

 Separately, I think the paper would do better by specifying the age of children being included in the analysis. Is this <18, 12-15, <12, etc as the analysis may vary based on this.

Does the article adequately reference differing views and opinions?

Yes

Are all factual statements correct, and are statements and arguments made adequately supported by citations?

Partly

Is the Open Letter written in accessible language?

Yes

Where applicable, are recommendations and next steps explained clearly for others to follow?

Yes

Is the rationale for the Open Letter provided in sufficient detail?

Yes

Reviewer Expertise:

Epidemiology, Clinical Medicine, HIV, Infectious Diseases

I confirm that I have read this submission and believe that I have an appropriate level of expertise to confirm that it is of an acceptable scientific standard, however I have significant reservations, as outlined above.

# Response

**Published:** 2021-11-22

**Authors:** Rachel Gur-Arie

**Affiliations:** Johns Hopkins University, USA

This article is focused on providing a synthesis of the ethics of COVID-19 vaccination for children.

 Overall, I think an evidence-based synthesis that it remains unclear even within communities to vaccinate all young children with the currently available vaccines and also that it is particularly challenging in the context of global vaccine inequities. The strengths are clear in that it takes a neutral tone and is data-driven. However, I believe it could be strengthened in a few ways.

- Thank you!

Since it is not a systematic review, I believe the way to maximize the potential impact of the work is to present it in the context of a broadly accepted ethical framework. It may be a single framework that evaluates the individual risk:benefit ratio of vaccination and then another one that evaluates it globally--or maybe the same framework if feasible. Either way, I think presenting it this way will go a long way in terms of the transition from this being a commentary to more of an ethical analysis.

- We agree with you that the best way to present our open letter is in the context of a broadly accepted ethical framework. The framework adopted in this open letter is the ethics framework of global public health ethics. As bioethicists, we recognize that the framework in the open letter’s first version is very implicit, and we have edited accordingly to make our framework of analysis more explicit, both in the introduction and in the last paragraph of the open letter. We also refer to the relevant public health ethics literature for a number of more specific arguments (e.g., in the “Protecting risk groups does not require vaccinating children” section).

At the individual level, I also think including the argument that is often used about even if there is some risk to children, the benefit of decreased onward transmission is worth it--i.e., that children potentially assume risk for population-level benefits. It would be helpful that the ethical analysis includes an assessment of this within broadly accepted ethical frameworks.

- Thank you for this suggestion, and we believe that our arguments in the “Protecting risk groups does not require vaccinating children” section support this claim. However, we have added your exact language to the beginning of the section to make this line of reasoning clearer.

Separately, I think the paper would do better by specifying the age of children being included in the analysis. Is this <18, 12-15, <12, etc as the analysis may vary based on this.

- Thank you for this clarification. We are conducting this analysis specifically for: “young healthy children (e.g., those aged under 15 with no co-morbidities), acknowledging that relevant considerations may change in older children or those with significant medical conditions” and have now specified that in the open letter.
